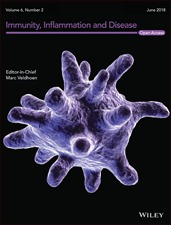# Issue Information

**DOI:** 10.1002/iid3.194

**Published:** 2018-05-11

## Abstract